# Ontogenetic development of auditory sensitivity and sound production in the squeaker catfish *Synodontis schoutedeni*

**DOI:** 10.1186/1741-7007-8-10

**Published:** 2010-01-29

**Authors:** Walter Lechner, Lidia Eva Wysocki, Friedrich Ladich

**Affiliations:** 1 University of Vienna, Department of Behavioural Biology, Althanstrasse 14, 1090 Vienna, Austria

## Abstract

**Background:**

Surveys of ontogenetic development of hearing and sound production in fish are scarce, and the ontogenetic development of acoustic communication has been investigated in only two fish species so far. Studies on the labyrinth fish *Trichopsis vittata *and the toadfish *Halobatrachus didactylus *show that the ability to detect conspecific sounds develops during growth. In otophysine fish, which are characterized by Weberian ossicles and improved hearing sensitivities, the ontogenetic development of sound communication has never been investigated. We analysed the ontogeny of the auditory sensitivity and vocalizations in the mochokid catfish *Synodontis schoutedeni*. Mochokid catfishes of the genus *Synodontis *are commonly called squeakers because they produce broadband stridulation sounds during abduction and adduction of pectoral fin spines. Fish from six different size groups - from 22 mm standard length to 126 mm - were studied. Hearing thresholds were measured between 50 Hz and 6 kHz using the auditory evoked potentials recording technique; stridulation sounds were recorded and their sound pressure levels determined. Finally, absolute sound power spectra were compared to auditory sensitivity curves within each size group.

**Results:**

The smallest juveniles showed the poorest hearing abilities of all size groups between 50 and 1,000 Hz and highest hearing sensitivity at 5 and 6 kHz. The duration of abduction and adduction sounds and the pulse period increased and sound pressure level (in animals smaller than 58 mm) increased, while the dominant frequency of sounds decreased with size in animals larger than 37 mm. Comparisons between audiograms and sound spectra revealed that the most sensitive frequencies correlate with the dominant frequencies of stridulation sounds in all *S. schoutedeni *size groups and that all specimens are able to detect sounds of all size groups.

**Conclusions:**

This study on the squeaker catfish *S. schoutedeni *is the first to demonstrate that absolute hearing sensitivity changes during ontogeny in an otophysine fish. This contrasts with prior studies on two cypriniform fish species in which no such change could be observed. Furthermore, *S. schoutedeni *can detect conspecific sounds at all stages of development, again contrasting with prior findings in fishes.

## Background

Fish possess a large diversity in hearing sensitivities. Several non-related groups of bony fish - often termed *hearing specialists *[1] - have evolved mechanisms which transmit vibrations of air-filled cavities to the inner ear. These mechanisms enable them to detect the pressure component of sound, enhance their absolute hearing sensitivity and broaden the range of detectable frequencies up to several kilohertz [2] versus several hundred Hertz in species without such specialization. Otophysi, a group of mainly freshwater fishes comprising the orders Siluriformes (catfishes), Cypriniformes (carps and loaches), Characiformes (tetras) and Gymnotiformes (South American knifefishes) are characterized by possessing such an accessory auditory structure, the Weberian apparatus. The Weberian apparatus consists of a chain of one to four ossicles that transmit oscillations of the swimbladder in the sound field directly to the inner ear. Catfishes, numbering more than 3,000 known species [3], show a high diversity in the structure of the Weberian apparatus and swimbladders. Their hearing ability depends on swimbladder size as well as on the number of Weberian ossicles [4].

Numerous studies have been conducted on the ontogenetic development of hearing and acoustic communication in mammals and birds [for example, [5-10]], but only a few have been carried out in fishes. Studies on the ontogeny of hearing in teleosts show varying results, ranging from no differences between two size groups of goldfish [11] and various size groups of the zebrafish *Danio rerio *[12], no change in absolute thresholds but expansion of the detectable frequency range in the zebrafish [13,14], up to an improvement of hearing abilities with size in the damselfish *Stegastes partitus *[15], the Red Sea bream *Pagrus major *[16], the labyrinth fish *T. vittata *[17] and the toadfishes *Porichthys notatus *[18] and *Halobatrachus didactylus *[19]. Egner and Mann [20] found a slight decrease in hearing sensitivity at lower frequencies during ontogeny of the damselfish *Abudefduf saxatilis*.

Ontogenetic development of sound production in fishes seems to follow a consistent pattern. Dominant frequencies of sounds decrease with size, for example in gurnards, mormyrids, croaking gouramis, damselfish and toadfish [17,19,21-26]. In most species tested in those studies, sound pressure levels, total duration of sounds as well as pulse periods within sounds increased.

The relationship between the development of hearing and sound production has been investigated in just two species so far. In both species - the anabantoid *Trichopsis vittata*, a hearing specialist, and the non-related batrachoidid *H. didactylus*, a hearing generalist - the smallest size groups were unable to detect conspecific sounds [17,19].

The present study is the first to investigate the ontogenetic development of hearing and sound production in an otophysine fish. The mochokid catfish *Synodontis schoutedeni *David 1936 emits stridulatory sounds in distress situations and during agonistic interactions by rubbing the spines of its pectoral fins in grooves of the shoulder girdle [27]. Therefore, mochokids are frequently called *squeakers*. *S. schoutedeni *shows a well-developed unpaired swimbladder, three Weberian ossicles and very good hearing sensitivities as compared to other species of catfish with a different swimbladder morphology [4]. We describe the developmental changes of temporal, spectral and intensity characteristics of stridulation sounds, analyse the development of auditory sensitivity with growth, and examine whether *S. schoutedeni *is able to communicate acoustically throughout its life history.

## Results

### Auditory sensitivity

**Figure 1:**
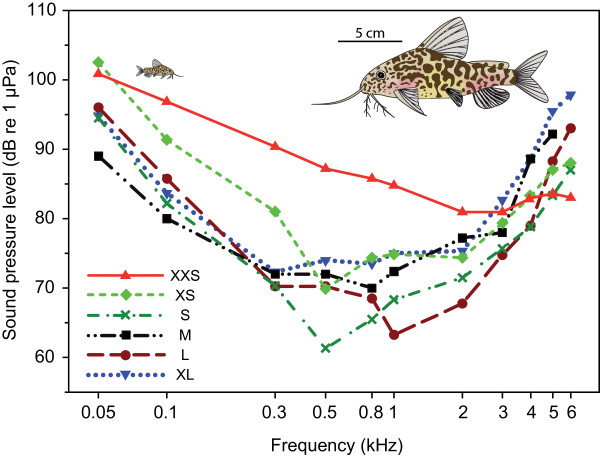
**Auditory evoked potential audiograms of the size groups investigated**. Mean hearing thresholds of the size groups XXS (N = 12), XS (N = 6), S (N = 6), M (N = 5), L (N = 4) and XL (N = 6) of *Synodontis schoutedeni *tested. Catfish pictures show representative specimens of group XXS (left) and XL (right) drawn in proportional scale for comparative purposes.

**Figure 2:**
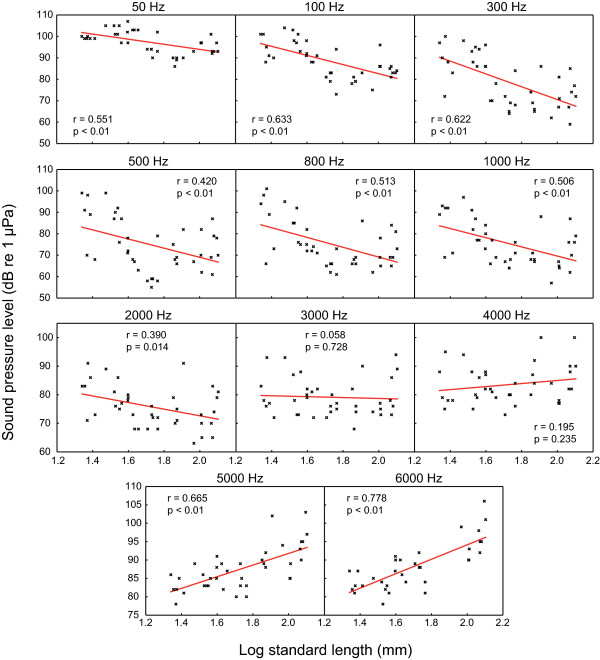
**Correlations between auditory thresholds and fish size at frequencies tested**. Semilog plots of hearing thresholds of each individual against log of standard-length at each frequency tested. N = 39 at each frequency except 6000 Hz (N = 34). Pearson's correlation coefficients and significances are given in graphs. Regression equations: x = log standard length, y = hearing threshold (dB re 1 μPa); 50 Hz: y = -1.92x + 117.79; 100 Hz: y = -21,33x + 125.27; 300 Hz: y = -29.91x + 130.33; 500 Hz: y = -21.32x + 111.67; 800 Hz: y = -22.84x + 114.81; 1000 Hz: y = -21.44x + 112.46; 2000 Hz: y = -11.57x + 95.77; 3000 Hz: y = -1.57x + 81.83; 4000 Hz: y = 5.34x + 74.30; 5000 Hz: y = 15.83x + 60.13; 6000 Hz: y = 19.68x + 54.78.

Auditory evoked potentials were recorded in all test groups between 50 Hz and 6 kHz. Best hearing abilities were shown at frequencies between 0.3 and 1 kHz in all size groups, except the smallest group XXS. The largest group XL showed its lowest hearing threshold at 0.3 kHz (72.3 dB re 1 μPa), group XXS at 2 and 3 kHz (80.9 dB) (Figure 1, Table 1). Group XXS and XS showed poorest hearing abilities of all groups at 0.05 kHz. Group XXS showed lowest hearing abilities of all groups from 0.1 to 2 kHz, but showed best hearing abilities of all groups at the highest frequency tested (6 kHz). Group S had well-developed hearing abilities at low frequencies similar to larger size groups, but hearing sensitivity similar to that of group XXS and XS at the highest frequencies tested (5 and 6 kHz) (Figure 1, Table 1). Significant correlations between size and hearing thresholds existed at most frequencies. At lower frequencies (0.05 to 2 kHz), larger animals showed significantly better hearing, whereas at the highest frequencies tested (5 and 6 kHz) the opposite was the case: smaller animals had lower hearing thresholds. At 3 and 4 kHz no correlation was evident (Figure 2).

**Table 1:** Hearing threshold values.

f (kHz)	XXS	XS	S	M	L	XL
**0.05**	100.83 +/- 0.79	102.50 +/- 1.31	94.50 +/- 1.63	89.00 +/- 0.77	96.00 +/- 1.00	94.83 +/- 1.42
**0.1**	96.83 +/- 1.56	91.33 +/- 1.50	82.17 +/- 2.81	80.00 +/- 0.89	85.75 +/- 4.29	83.67 +/- 0.71
**0.3**	90.33 +/- 2.23	81.00 +/- 4.19	70.33 +/- 2.97	72.00 +/- 3.83	70.25 +/- 4.03	72.33 +/- 3.61
**0.5**	87.17 +/- 3.06	69.83 +/- 2.09	61.33 +/- 3.58	72.00 +/- 2.92	70.25 +/- 4.25	74.00 +/- 3.74
**0.8**	85.75 +/- 3.20	74.33 +/- 1.65	65.50 +/- 1.73	70.00 +/- 3.26	68.50 +/- 3.48	73.50 +/- 3.05
**1**	84.75 +/- 2.68	74.83 +/- 2.77	68.33 +/- 1.87	72.40 +/- 3.96	63.25 +/- 2.17	75.00 +/- 3.44
**2**	80.92 +/- 1.87	74.33 +/- 2.23	71.50 +/- 1.26	77.20 +/- 3.80	67.75 +/- 2.29	75.33 +/- 2.84
**3**	80.92 +/- 2.21	79.33 +/- 1.54	75.67 +/- 1.12	78.00 +/- 3.74	74.75 +/- 0.85	82.67 +/- 3.36
**4**	82.92 +/- 2.03	83.17 +/- 1.45	78.83 +/- 1.56	88.60 +/- 3.46	79.00 +/- 1.68	88.67 +/- 2.72
**5**	83.58 +/- 0.82	87.00 +/- 1.29	83.33 +/- 1.38	92.20 +/- 2.54	88.25 +/- 2.14	95.50 +/- 1.78
**6**	83.00 +/- 0.74	88.00 +/- 1.13	87.00 +/- 1.59	-	93.00 +/- 2.12	97.83 +/- 2.06

Mean hearing threshold values (+/- s.e.m., dB re 1 μPa) of the six size groups of *S. schoutedeni *at each frequency tested. f = frequency; for exact size ranges see material and methods; group M was not tested at 6 kHz.

### Sound production

**Figure 3:**
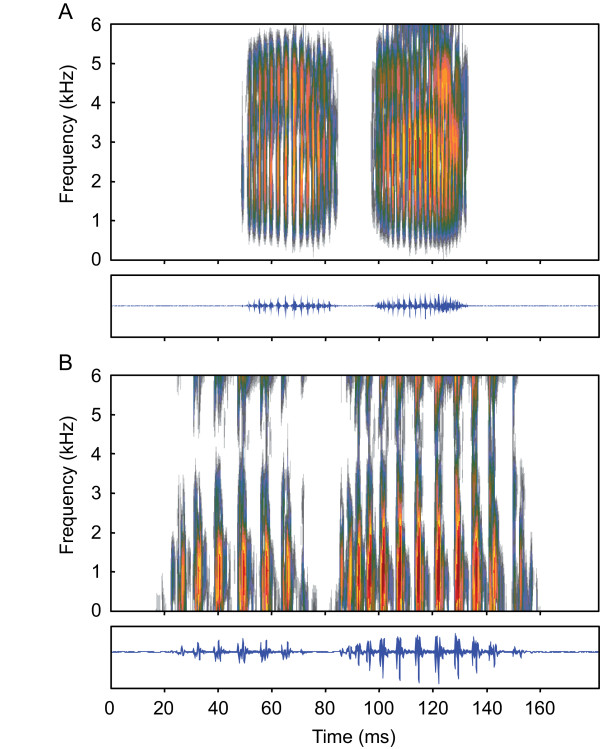
**Sonagram and oscillogram of stridulation sounds**. Sonagram (top) and oscillogram (below) of adduction sounds (left) and abduction sounds (right) of representatives of group XXS **(A) **and group XL **(B)**. Sampling frequency 44.1 kHz, filter bandwidth 650 Hz for XXS and 600 Hz for XL, 75% overlap, Hanning window.

**Figure 4:**
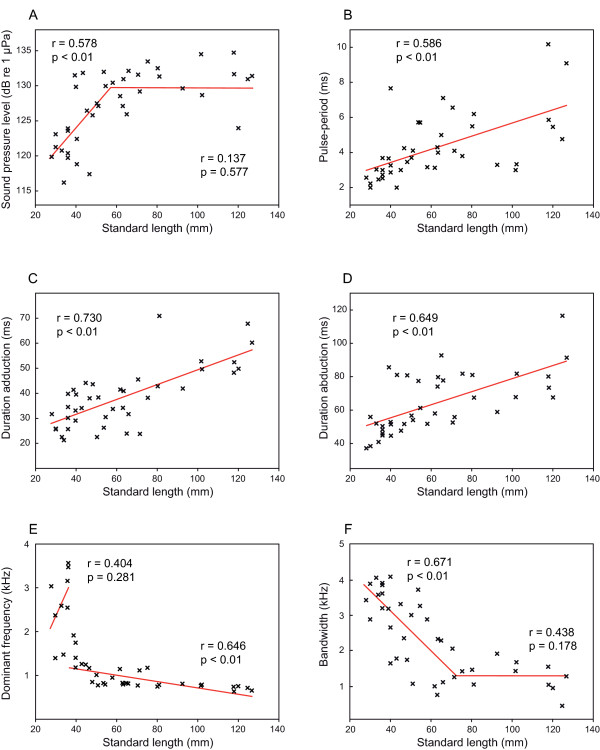
**Correlations between sound characteristics and standard length**. Plots of sound characteristics against standard length. Pearson's correlation coefficients and significances are given in graphs. **4A**: Segmented linear regression plot SPL against SL, breaking point 57.6 mm SL, regression equations (y = SPL): SL < 57.6 mm: N = 21, SPL = 0.36 SL + 109.76, SL > 57.6 mm: N = 19, SPL = 0.02 SL + 129.10; **4B**: Pulse-period (adduction) against SL: N = 40, pulse period = 0.04 SL + 1.93; **4C**: Duration of adduction sounds against SL: N = 40, duration = 0.30 SL + 19.59; **4D**: Duration of abduction sounds against SL: N = 40, duration = 0.39 SL + 39.69; **4E**: Segmented linear regression plot of dominant frequency against SL, breaking point 36.91 mm SL: SL < 36.91 mm: N = 9, dom. freq. = 101.35 SL - 741.89, SL > 36.91 mm: N = 31, dom. freq. = - 7.04 SL + 1469.41; **4F**: Segmented linear regression plot bandwidth of sounds 10 dB below SPL of peak frequency against SL, breaking point 73.43 mm SL: SL < 73.43 mm: N = 24, bandwidth = - 53.42 SL + 5174.77, SL > 73.43 mm: N = 11, bandwidth = - 9.36 SL + 2210.15. Regression lines in 4A, 4E and 4F were drawn according to the results of the segmented linear regression calculation. Note two p and r values (one for each regression) in figures 4A, 4E and 4F.

Stridulatory sounds were emitted during abduction (forward movement) and adduction (backward movement) of pectoral spines in all groups tested as soon as specimens were handled (Figure 3). Swimbladder drumming sounds could not be detected or recorded. Sound pressure level (SPL) increased with size up to 58 mm standard length (SL) whereas no further increase was observed in larger-sized animals. SPLs of animals up to 58 mm SL were significantly lower than in larger specimens (Mann-Whitney-U test: U = 62.5, N = 40, *P *< 0.01). In larger fish, no further increase in SPL was evident (Figure 4A). Duration of adduction and abduction sound as well as pulse period (PP) increased with size (Figures 3 and 4B-D). The dominant frequency of sounds decreased significantly with size in fish larger than 37 mm SL, while no correlation was found in smaller ones (Figure 4E). However, the dominant frequency of fish up to 37 mm SL was significantly higher than in larger fish (Mann-Whitney-U test: U = 5, N = 40, *P *< 0.01). While bandwidth decreased with increasing size in fish up to 73 mm SL, no further decrease of bandwidth was observed in larger fish. The main energies of stridulation sounds produced by smaller specimens were more broad-banded than those of larger ones (Mann-Whitney-U test: U = 44, N = 40, *P *< 0.01; Figures 3 and 4F).

### Comparison between audiograms and sound spectra

**Figure 5:**
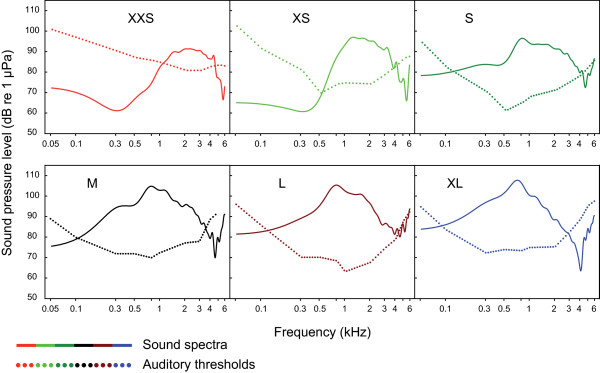
**Relations between auditory thresholds and sound spectra**. Cepstrum-smoothed sound power spectra of stridulatory sounds calculated for a distance of 5 cm from the hydrophone compared to auditory thresholds in the six size groups tested. For size-ranges see material and methods.

All size groups showed best hearing abilities in frequency ranges where main energies of stridulation sounds were concentrated, and all size groups were able to detect conspecific sounds (Figure 5). Thus, group XL detected sounds produced by group XXS and vice versa.

## Discussion

### Development of hearing

Changes in hearing abilities have been reported in several fish taxa [15,16,18,19]. The present study provides the first evidence that auditory thresholds change during ontogeny in an otophysine fish species (Figure 2), results that are in contrast with previous studies on two species of cypriniforms, namely the goldfish [11] and the zebrafish [14]. Several potential explanations can be forwarded for this discrepancy among otophysines. First, different species have been investigated, which even belong to different orders, namely Cypriniformes and Siluriformes. Furthermore, the authors of those studies did not calculate regressions of hearing abilities including all data from each individual study specimen, but instead compared the mean results of different size groups. Furthermore, the size differences of the goldfish used as well as the range of frequencies tested might have been too small: the specimens were 45 to 48 mm SL and 110 to 120 mm SL, and the five test frequencies ranged from 100 to 2,000 Hz. However, goldfish are able to detect sounds at least up to 4 kHz [28]. In contrast to the goldfish study, Higgs et al. measured early stages of zebrafish (10 mm) [14] and found an extension of the maximum frequency detectable from a 200 Hz upper limit in small specimens up to 4 kHz in large ones but no change in absolute thresholds. They argued that the development of the Weberian ossicles is responsible for this increase in the detectable frequency range. By contrast, Zeddies and Fay [12] found in their study on the development of startle response in zebrafish no change of stimulus thresholds and frequency bandwidth to which the zebrafish responded from five days post fertilization to adult. Similar to the observations of Zeddies and Fay [12] in zebrafish, we did not observe a change in the range of detectable frequencies. Based on Coburn and Grubach's [29] study on the ontogeny of the Weberian ossicles in several species of catfish we assume that all our tested animals possessed fully developed Weberian ossicles. Thus, the current study is the first to systematically demonstrate that auditory thresholds change with size in an otophysine fish (Figure 2), whereas the detectable frequency range does not change (Figures 1 and 2). Auditory sensitivity increased at lower frequencies up to 2 kHz and decreased at 5 and 6 kHz.

Some prior data on other catfish species are in agreement with these findings. In eight specimens of the loricariid catfish *Ancistrus ranunculus*, the four smallest specimens tested detected sound stimuli at 5 kHz, whereas only one of the larger ones responded to 5 kHz stimuli at the maximum SPL tested (129 dB re 1 μPa) [4]. This indicates that smaller individuals of this loricariid had higher auditory sensitivities at the highest frequency tested. A similar observation can be made when comparing results of three studies on the pimelodid catfish *Pimelodus pictus *[30-32]. The smallest specimens tested by Wysocki et al. [32] had better hearing abilities at the highest frequencies tested (mean hearing threshold at 4 kHz: 75.3 dB re 1 μPa) than the largest fish tested by Ladich [30] (81.3 dB).

These data indicate that smaller individuals within a given species of catfish may hear better at higher frequencies. One possible reason could be that smaller specimens produce sounds of higher frequencies and are adapted to detect sounds of similar-sized conspecifics. Ladich and Yan [33] argued that the pygmy gourami, the smallest species investigated in their comparative study on labyrinth fishes (family Osphronemidae), heard better at 3 to 5 kHz than the larger two species. The authors argued that higher sensitivity at higher frequencies in the smallest species may reflect the higher resonance frequencies of their smaller-sized accessory hearing structures, the so-called suprabranchial chambers (paired air-filled chambers close to the inner ears).

Studies on the ontogenetic development of hearing in Perciformes (Pomacentridae - damselfishes, Osphronemidae - gouramis, Sparidae - sea breams) and Batrachoidiformes (Batrachoididae - toadfishes) [15-19] show a more consistent pattern. They reported mostly a moderate improvement of hearing with size, with one exception. Kenyon [15] observed a considerable 60 dB increase in hearing sensitivity in the bicolor damselfish *Stegastes *(syn. *Pomacentrus*) *partitus *at the most sensitive frequency (500 Hz) during ontogeny. Contrary to Kenyon's results, Egner and Mann [20] reported a decrease of hearing sensitivity in a representative of the same family. The sensitivity decreased in the sergeant major damselfish *Abudefduf saxatilis *with growth at 100 and 200 Hz. Iwashita et al. [16] observed an increase in hearing sensitivity in zero-, one- and two-year-old red sea bream *Pagrus major*, in particular between 100 and 300 Hz. In the croaking gourami *Trichopsis vittata*, different trends were found at different frequencies during development of hearing [17]. Hearing improved with size in the frequency range from 800 to 2,000 Hz, where the main energies of sounds were concentrated. An opposite trend was observed at 4 and 5 kHz. This decrease in sensitivity at highest frequencies in *T. vittata *is in agreement with the catfish data of the current study. In both toadfish species tested so far, the plainfin midshipman [18] and the Lusitanian toadfish [19], a small increase of hearing sensitivity with size was evident. In the Lusitanian toadfish, hearing improved with growth at three out of seven frequencies tested.

### Development of sound production

Whereas different trends were found in the development of auditory sensitivities in different species, the development of sound characteristics shows more similar patterns among teleosts. These patterns agree with the current data in *Synodontis schoutedeni*.

The dominant frequency of sounds is negatively correlated with body size in representatives of all families investigated so far, for example, in pomacentrids - *Stegastes partitus*, *Dascyllus albisella, Amphiprion akallopisos, A. clarkii, A. frenatus*, *A. ocellaris*, [23,26,34,35], osphronemids - *Trichopsis vittata*, *T. pumila *and *T. schalleri *[17,21,22], sciaenids - *Cynoscion regalis *[36], triglids - *Eutrigla gurnardus *[25], and toadfishes - *Halobatrachus didactylus *[19]. Fine et al. [37] found a decrease of center frequency in the ictalurid catfish *Ictalurus punctatus*. In contrast, Ladich [38] reported such a correlation in only one out of six catfish species from three families. Only in the doradid *Platydoras armatulus *(formerly named *P. costatus*) did the dominant frequency decrease with size, while no such correlation was present in pimelodids and mochokids. The lack of a relationship might have been due to the difficulty of determining dominant frequencies in broad-band sounds and perhaps the small size differences of fish used in that study. In the present investigation, *S. schoutedeni *size differed more (1.02 g to 51.80 g vs. 1.7 to 5.6 g in [38]). A significant correlation between dominant frequency of sounds and size was found in animals larger than 37 mm SL. The decrease in dominant frequency may be an effect of longer pulse periods within sounds, and thus based on size-related changes in morphology [36]. However, these underlying structural changes seem not to apply to animals smaller than 37 mm in which dominant frequencies were significantly higher than expected by the linear regression. Smaller individuals of *S. schoutedeni *showed a more evenly distributed sound energy than large individuals. The frequency band 10 dB below the dominant frequency ranged from ca. 2,800 to 4,100 Hz in smallest fish down to ca. 370 to 1,500 Hz in largest ones (Figures 3 and 4F). The decrease in bandwidth was significant from the smallest size group up to 73 mm SL; no further change was observed in larger animals. No such relationship has been described in other species so far, although a closer examination of sounds of other species might yield similar results. The power spectra of sounds of three species of clownfishes *Amphiprion *spp. [26] indicate that such a relationship may also exist in other fish species.

The sound pressure level (SPL) of vocalizations increases with size in species from different taxa such as in the tigerfish *Therapon jarbua *[39], the osphronemid *T. vittata *[17], the sciaenid *C. regalis *[36], the toadfish *H. didactylus *[19], and in the mochokid catfish *S. schoutedeni *(current study). Interestingly, while SPLs increased rapidly in animals below 58 mm SL, no further increase of SPLs occurred in the larger specimens (Figure 4A). The amplitude of sounds depends on anatomical constraints and on how long and hard fish press the dorsal process of the pectoral spine against the groove of the shoulder girdle [37,40]. This could cause intraindividual variation of SPLs and a lack of correlation in larger animals.

Temporal characteristics of sounds such as duration of abduction and adduction sounds and pulse period of sounds increase with size in *S. schoutedeni*. This can be explained by the growth of the dorsal process of the pectoral spine and the fact that a full pectoral sweep takes longer in larger fish than in a smaller one [37,40]. Increases with size have also been observed for pulse period in the Lusitanian toadfish [19], pulse duration in weakfish [36], and grunt duration in the grey gurnard [25].

### Comparison between hearing abilities and spectra of stridulation sounds

In all tested size groups of *S. schoutedeni*, the main energies of stridulation sounds correspond to the most sensitive frequency range of hearing (Figure 5). Despite some uncertainty of sound level measurements in aquaria as compared to free field conditions our data show that all groups are apparently able to detect sounds produced by specimens of the other groups, which is in contrast to prior findings in other teleost species. In croaking gouramis and Lusitanian toadfish, individuals of the smallest size groups were probably unable to detect sounds produced by similar-sized conspecifics [17,19] based on a comparison between absolute sound spectra and audiograms at a communication distance of 3 to 10 cm. The reason for this difference between *S. schoutedeni *and the latter two perciform species is two-fold: First, *S. schoutedeni *shows much higher hearing sensitivities than the other two species investigated, namely the croaking gourami and the Lusitanian toadfish [17,19]. Early stages of the mochokid catfish are more sensitive at the dominant frequencies of conspecific sounds than in the other two species. Secondly, the SPLs of juveniles' sounds are much higher than those of juveniles of the other two species. Thus, all squeaker catfish can detect sounds of conspecifics either uttered during agonistic intraspecific interactions or in a distress context [41].

## Conclusions

The current study shows varied changes in auditory sensitivity during the ontogenetic development of *S. schoutedeni*. Hearing sensitivities increase with growth in the frequency range where main energies of sounds are concentrated, whereas the opposite is the case in the highest tested frequencies (5 and 6 kHz). The ontogenetic development of sounds follows fish-typical patterns, namely an increase in sound level and duration, and a decrease in dominant frequency of sounds. Contrary to previous studies, the present data show that the squeaker catfish can communicate with conspecifics of all size groups at short distances either during agonistic encounters or when being attacked by predators. These enhanced sound-detecting abilities of otophysines enable catfish to orient and communicate acoustically at much earlier stages of development.

## Methods

### Animals

For hearing measurements, fish were grouped into six size groups, XXS (SL = 21.9 to 36.5 mm, N = 12), XS (SL = 39.5 to 45.3 mm, N = 6), S (SL = 51.0 to 58.1 mm, N = 6), M (SL = 71.1 to 81.0 mm, N = 5), L (SL = 92.6 to 102.6 mm, N = 4) and XL (SL = 116.0 to 126.8 mm, N = 6). Hearing thresholds of group M were taken from Lechner and Ladich [4]. For sound recordings, corresponding groups were defined. Because not every specimen vocalized during the sound recording procedures, minimum and maximum size ranges differed slightly: XXS: SL = 28.0 to 36.0 mm, N = 9; XS: SL = 39.0 to 45.0 mm, N = 6; S: SL = 46.7 to 58.1 mm, N = 7; M: SL = 61.8 to 81.1 mm, N = 10; L: SL = 92.6 to 102.2 mm, N = 3; XL: SL = 117.8 to 126.8 mm, N = 5.

Fishes of groups M (hearing tests) to XL were wild caught from Malebo Pool (Congo River, Democratic Republic of the Congo) and obtained from a tropical fish supplier (Transfish, Munich, Germany). After a quarantine of three weeks, fish were acclimated to our aquaria for at least two months prior to the first experiments. Specimens of groups XXS, XS, S and the specimens of group M used for sound recordings were aquarium reared. For a detailed description of the breeding procedure (breeding occurred spontaneously and was not induced by hormone injections) of the tested specimens see Drescher [42]. Because breeding and rearing mochokid catfishes in aquaria is very difficult, we had only a small number of offspring and we were also interested in measuring fish of later stages, we did not test any specimens smaller than 22 mm SL.

Fish were kept in planted aquaria with a sand bottom equipped with roots and clay or bamboo tubes as shelters. Only external filters were used. In order to provide a quiet environment, we did not use any internal filters or air stones. Temperature was kept at 25 ± 1°C and a 12 h: 12 h L: D cycle was maintained. Fish were fed frozen chironomid larvae and artificial food (granulate, flakes and tablets); the small specimens of groups XXS, XS and S were also fed Cyclop-Eeze^® ^(freeze-dried copepods, Argent Chemical Laboratories, Redmond, WA, USA). Since fry and juveniles grow very unequally despite identical conditions of husbandry [13,43], we classified the tested specimens as different size groups rather than age groups. Standard length (SL) was measured as *standard length 2 *following Holcik et al. [44]. Using total length or body mass instead of SL for analyses did not change the results.

All experiments were performed with the permission of the Austrian Federal Ministry for Education, Science and Culture (GZ 66.006/2-BrGT/2006).

### Auditory sensitivity measurements

Hearing thresholds were obtained using the AEP recording technique developed by Kenyon et al. [28] and modified by Wysocki and Ladich [45,46]. Only a brief description of the technique is given here. Test subjects were mildly immobilized with Flaxedil (gallamine triethiodide; Sigma-Aldrich, Vienna, Austria) diluted in a Ringer solution. The dosage applied (3.5 to 5.4 μg g^-1^) allowed fish to still perform slight opercular movements but not to initiate significant myogenic noise that could interfere with the AEP recordings. All auditory measurements were carried out in a bowl-shaped plastic tub (diameter 33 cm, water depth 13 cm, 1 cm layer of gravel), which was lined inside with acoustically absorbent material (air-filled packing wrap) to decrease resonances and reflections [47]. The tub was positioned on an air table (TMC Micro-g 63-540, Technical Manufacturing Corporation, Peabody, MA, USA), which rested on a vibration-isolated plate of concrete. A sound-proof chamber, constructed as a Faraday cage (interior dimensions: 3.2 m × 3.2 m × 2.4 m), enclosed the whole setup. The subjects were placed at the water surface in the center of the tub. The contacting points of the electrodes were maximally 1 to 2 mm above the water surface. Tissue paper (Kimwipes^®^, Kimberly-Clark Corporation, Irving, TX, USA) was placed on the fish head to keep it moist and ensure proper contact of electrodes. Respiration was achieved through a temperature-controlled (25 ± 1°C), gravity-fed water circulation system using a pipette inserted into the subject's mouth. The AEPs were recorded by using silver wire electrodes (0.38 mm diameter) pressed firmly against the skin: the recording electrode was placed over the region of the medulla and the reference electrode cranially between the nares. Shielded electrode leads were attached to the differential input of an AC preamplifier (Grass P-55, Grass Technologies, West Warwick, RI, USA, gain 100 x, high-pass at 30 Hz, low-pass at 1 kHz), with a ground electrode placed in the water near the fish's body. A hydrophone (Brüel and Kjaer 8101, Naerum, Denmark; frequency range 1 Hz to 80 kHz ± 2 dB; voltage sensitivity -184 dB re 1 V μPa^-1^) was placed close to the head on the right side of the animals (approximately 1 cm away) in order to determine absolute stimulus SPLs. A custom-built preamplifier was used to boost the hydrophone signal. Both presentation of sound stimuli and AEP waveform recording were achieved using a modular rack-mount system (Tucker-Davis Technologies (TDT) System 3, Gainesville, FL, USA) controlled by a PC containing a TDT digital signal processing board and running TDT BioSig RP software (Tucker-Davis Technologies, Gainesville, FL, USA).

### Presentation of sound stimuli

Hearing thresholds were determined at 0.05, 0.1, 0.3, 0.5, 0.8, 1, 2, 3, 4, 5 and 6 kHz. The duration of sound stimuli increased from two cycles at 50 Hz and 100 Hz up to eight cycles at 4 kHz and above. Rise and fall times increased from one cycle at 50 to 300 Hz up to three cycles at frequencies from 2 to 6 kHz. All bursts were gated using a Blackman window. Data for group M were taken from Lechner and Ladich [4], and this group was not tested at 6 kHz. For each test condition, one thousand stimuli were presented at opposite polarities, that is, 90° and 270°, and were averaged together by the BioSig RP Software, yielding a 2000-stimulus trace to eliminate any stimulus artefact. The SPL was reduced in 4-dB steps. Close to hearing threshold, this procedure was performed twice and the AEP traces were overlaid to visually check if they were repeatable. The lowest SPL at which a repeatable AEP trace could be obtained, as determined by overlaying replicate traces, was defined as the threshold [see also [48]]. Sound stimuli waveforms were created using TDT SigGen RP software (Tucker-Davis Technologies, Gainesville, FL, USA). Tone-bursts were presented through two speakers (Fostex 256 PM-0.5 Sub and PM-0.5 MKII, Fostex Corporation, Tokyo, Japan). These were positioned 0.5 m above the water surface.

### Sound recordings and sound pressure level measurements

The experiments were performed in a test tank (50 cm × 27 cm × 30 cm; length × width × height; water depth 25 cm) whose bottom was covered with sand. The aquarium walls, except for the front glass, were lined on the inside with air-filled packing wrap in order to reduce resonances and reflections. Prior empirical tests using white noise and pulsed sounds had shown that lining reduced artefacts such a high frequency resonances, which are a known phenomenon of small tank acoustics in this setup [17]. Akamatsu et al. [[49], see their Figure 7c] and our own recordings (Figure 5) revealed resonance frequencies at 4 kHz and higher frequencies. Video and audio signals were stored synchronously on the hard disc of a DVD hard disc recorder (Panasonic DMR-EX95V, Panasonic Corporation, Osaka, Japan). A hydrophone (Brüel & Kjaer 8106, sensitivity -174 dB re 1 V per l Pa) was positioned about 5 cm off the center of the aquarium and connected to a microphone power supply (Brüel & Kjaer 2804) whose output was sent to the hard disc recorder. Simultaneous video recordings of fin movements were carried out by a video camera (Sony CCD-VX1E, Sony Corporation, Shinagawa, Tokyo, Japan) connected to the DVD hard disc recorder. Sound pressure levels (SPLs) were measured in parallel with the sound recordings, with the hydrophone power supply additionally connected to a sound level meter (Brüel & Kjaer Mediator 2238). Fish were held about 5 cm away from the hydrophone in the middle of the tank, with one pectoral fin fixed either by the frame of a fish net or the fingers of the testing person to avoid overlap of sounds produced by the left and right pectoral fins. According to Akamatsu et al. [49] a short recording distance reduces the effects of tank acoustics on SPLs and signal distortions. Sounds produced by both fins were recorded in each fish. All experiments were carried out in a walk-in soundproof room, which was constructed as a Faraday cage. Test tanks were placed on a vibration-isolated floor.

### Sound analysis

The VLC Media Player (VideoLAN, Club VIA, Châtenay Malabry, France, released under the GNU General Public License) was used to assign single sounds to right and left fins and to either abduction (of the body) or adduction (to the body) movement of the fins. All sounds were sampled at 44.1 kHz. Temporal characteristics of sounds were analysed using Raven Pro 1.3 for Windows (Bioacoustics Research Program, Cornell Laboratory of Ornithology, Ithaca, NY, USA) and spectral characteristics were analysed using STx 3.7 (Acoustics Research Institute, Austrian Academy of Sciences, Vienna, Austria).

**Figure 6:**
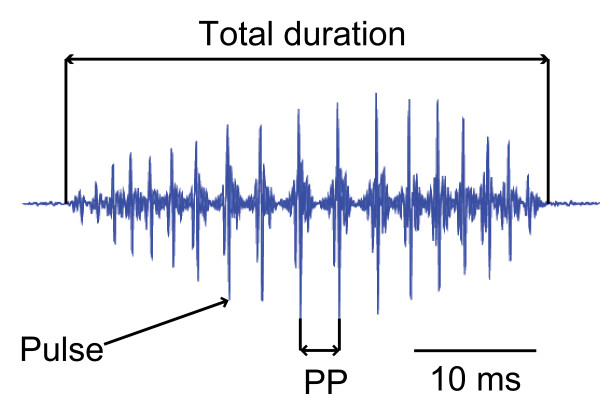
**Temporal sound characteristics of single calls**. Oscillogram of a single stridulation sound of *S. schoutedeni *showing temporal sound characteristics analysed (PP = pulse period).

**Figure 7:**
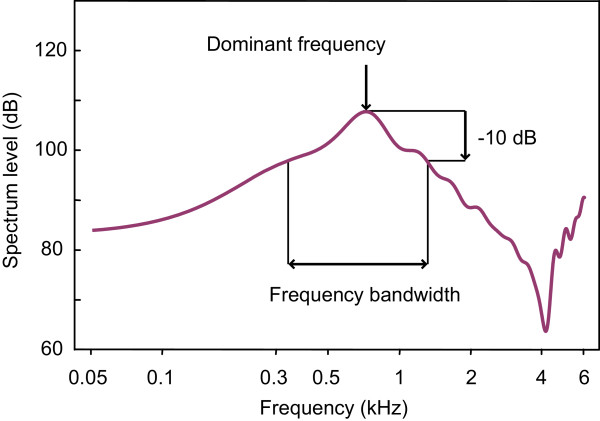
**Cepstrum-smoothed power spectrum of stridulatory sounds of a size group indicating the calculation of the frequency bandwidth used for analysis**. The minimum and maximum frequencies 10 dB below the dominant frequency were determined and the bandwidth calculated.

Ten abduction sounds and 10 adduction sounds were analysed per individual with respect to total duration and pulse period (except for four specimens of group XS, four specimens of group S and one specimen of group M, where only one to five abduction and adduction sounds could be analysed because of a lack of vocalising activity in these individuals) (Figure 6). The dominant frequency of sounds for each individual was determined from the sound power spectrum calculated from all stridulatory sounds emitted by one specimen. A sound file made up of vocalisations emitted by all specimens of a size group was created separately to calculate group-specific cepstrum-smoothed sound spectra [50]. Absolute sound spectra of the recordings were calculated as described previously [46,51]. A value *bandwidth -10 dB *was calculated for each size group, characterising the frequency range of sounds at a level 10 dB below the spectral sound level of the dominant frequency (Figure 7).

### Statistical analyses

All data were tested for normal distribution utilising Shapiro-Wilk's test. When data were normally distributed, parametric statistical tests were applied. Mean hearing thresholds were determined for each size group and at each frequency, and audiograms were drawn using SigmaPlot 10.0 (Systat Software/Cranes Software Inc., Bangalore, India and San Jose, CA, USA). Means of sound characteristics were calculated for each fish and used for further analyses. Relationships between fish size (SL or logSL) and hearing thresholds or sound characteristics were determined by Pearson's correlation coefficients and linear regressions. The statistical tests were performed with the software SPSS 17.0 (SPSS Inc., Chicago, IL, USA). If it was evident that data points in a graph showed two different distribution patterns, segmented linear regressions and breakpoints were calculated with the software SegReg (R. J. Oosterbaan, Wageningen, The Netherlands).

## Abbreviations

AEP: auditory evoked potentials; dom. freq.: dominant frequency; DVD: digital versatile disc; L: D cycle: light: dark cycle; L: large; M: medium; PP: pulse period; S: small; SL: standard length; SPL: sound pressure level, XL: extra large; XS: extra small; XXS: extra extra small.

## Authors' contributions

FL and WL conceived the study. WL did measurements and analysis of auditory sensitivity. WL and LEW recorded the sounds, WL and FL analysed them. WL, LEW and FL did statistical analyses and wrote the paper. WL prepared the figures and graphs. All authors read and approved the final manuscript.

## References

[B1] Hawkins AD Myrberg AA Jr Lewis B Hearing and sound communication underwater Bioacoustics, a Comparative Approach 1983 London: Academic Press 347 405

[B2] Popper AN Fay RR Fay RR, Popper AN The auditory periphery in fishes Comparative Hearing: Fish and Amphibians 1999 New York: Springer Verlag 43 100

[B3] Ferraris CJ Jr Checklist of catfishes, recent and fossil (Osteichthyes: Siluriformes), and catalogue of siluriform primary types Zootaxa 2007 1418 1 628

[B4] Lechner W Ladich F Size matters: diversity in swimbladders and Weberian ossicles affects hearing in catfishes J Exp Biol 2008 211 1681 1689 10.1242/jeb.016436 18456895

[B5] Dmitrieva LP Gottlieb G Development of brainstem auditory pathway in mallard duck embryos and hatchlings J Comp Physiol A Neuroethol Sens Neural Behav Physiol 1992 171 665 671 10.1007/BF00194114 1494141

[B6] Ehret G Romand R Development of hearing and response behavior to sound stimuli: behavioral studies Development of auditory and vestibular systems 1983 New York: Academic Press 211 237

[B7] Ehret G Romand R Postnatal development of absolute auditory thresholds in kittens J Comp Physiol Psychol 1981 95 304 311 10.1037/h0077770 7229161

[B8] Gray L Rubel EW Development of absolute thresholds in chickens J Acoust Soc Am 1985 77 1162 1172 10.1121/1.392180 3980868

[B9] Moss CF Redish D Gounden C Kunz TH Ontogeny of vocal signals in the little brown bat, *Myotis lucifugus* Anim Behav 1997 54 131 141 10.1006/anbe.1996.0410 9268443

[B10] Podos J Sherer JK Peters S Nowicki S Ontogeny of vocal tract movements during song production in song sparrows Anim Behav 1995 50 1287 1296 10.1016/0003-3472(95)80044-1

[B11] Popper AN The effects of size on auditory capacities of the goldfish J Aud Res 1971 11 239 247

[B12] Zeddies DG Fay RR Development of the acoustically evoked behavioral response in zebrafish to pure tones J Exp Biol 2005 208 1363 1372 10.1242/jeb.01534 15781896

[B13] Higgs DM Souza MJ Wilkins HR Presson JC Popper AN Age- and size related changes in the inner ear and hearing ability of the adult zebrafish (*Danio rerio*) JARO 2001 3 174 184 10.1007/s101620020035 PMC3202399 12162367

[B14] Higgs DM Rollo AK Souza MJ Popper AN Development of form and function in peripheral auditory structures of the zebrafish (*Danio rerio*) J Acoust Soc Am 2003 113 1145 1154 10.1121/1.1536185 12597208

[B15] Kenyon TN Ontogenetic changes in the auditory sensitivity of damselfishes (Pomacentridae) J Comp Physiol A Neuroethol Sens Neural Behav Physiol 1996 179 553 561

[B16] Iwashita A Sakamoto M Kojima T Watanabe Y Soeda H Growth effects on the auditory threshold of Red Sea bream Nippon Suisan Gakkaishi 1999 65 833 838

[B17] Wysocki LE Ladich F The ontogenetic development of auditory sensitivity, vocalisation and acoustic communication in the labyrinth fish *Trichopsis vittata* J Comp Physiol A Neuroethol Sens Neural Behav Physiol 2001 187 177 187 10.1007/s003590100186 11401197

[B18] Sisneros JA Bass AH Ontogenetic changes in the response properties of individual, primary auditory afferents in the vocal plainfin midshipman fish *Porichthys notatus *Girard J Exp Biol 2005 208 3121 3131 10.1242/jeb.01742 16081610

[B19] Vasconcelos RO Ladich F Development of vocalization, auditory sensitivity and acoustic communication in the Lusitanian toadfish *Halobatrachus didactylus* J Exp Biol 2008 211 502 509 10.1242/jeb.008474 18245626

[B20] Egner SA Mann DA Auditory sensitivity of sergeant major damselfish *Abudefduf saxatilis *from post-settlement juvenile to adult Mar Ecol-Prog Ser 2005 285 213 222 10.3354/meps285213

[B21] Ladich F Bischof C Schleinzer G Fuchs A Intra- and interspecific differences in agonistc vocalization in croaking gouramis (Genus: *Trichopsis*, Anabantoidei, Teleostei) Bioacoustics 1992 4 131 141

[B22] Henglmüller SM Ladich F Development of agonistic behaviour and vocalization in croaking gourami J Fish Biol 1999 54 380 395 10.1111/j.1095-8649.1999.tb00837.x

[B23] Myrberg AA Ha SJ Shamblott HS The sounds of bicolor damselfish (*Pomacentrus partitus*): predictors of body size and a spectral basis for individual recognition and assessment J Acoust Soc Am 1993 94 3067 3070 10.1121/1.407267

[B24] Crawford JD Hearing and acoustic communication in mormyrid electric fishes Mar Freshw Behav Physiol 1997 29 65 86 10.1080/10236249709379001

[B25] Amorim MCP Hawkins AD Ontogeny of acoustic and feeding behaviour in the Grey Gurnard, *Eutrigla gurnardus* Ethology 2005 111 255 269 10.1111/j.1439-0310.2004.01061.x

[B26] Parmentier E Colleye O Mann D Hearing ability in three clownfish species J Exp Biol 2009 212 2023 2026 10.1242/jeb.030270 19525428

[B27] Kaatz IM The behavioral and morphological diversity of acoustic communication systems in a clade of tropical catfishes (Pisces: Siluriformes) PhD thesis 1999 State University of New York, Syracuse, NY

[B28] Kenyon TN Ladich F Yan HY A comparative study of hearing ability in fishes: the auditory brainstem response approach J Comp Physiol A Neuroethol Sens Neural Behav Physiol 1998 182 307 318 10.1007/s003590050181 9528109

[B29] Coburn MM Grubach PG Ontogeny of the Weberian Apparatus in the armored catfish *Corydoras paleatus *(Siluriformes: Callichthyidae) Copeia 1998 1998 301 311 10.2307/1447426

[B30] Ladich F Did auditory sensitivity and vocalization evolve independently in otophysan fishes? Brain Behav Evol 1999 53 288 304 10.1159/000006600 10473905

[B31] Amoser S Ladich F Diversity in noise-induced temporary hearing loss in otophysine fishes J Acoust Soc Am 2003 113 2170 2179 10.1121/1.1557212 12703727

[B32] Wysocki LE Montey K Popper AN The influence of ambient temperature and thermal acclimation on hearing in a eurythermal and a stenothermal otophysan fish J Exp Biol 2009 212 3091 3099 10.1242/jeb.033274 19749101

[B33] Ladich F Yan HY Correlation between auditory sensitivity and vocalization in anabantoid fishes J Comp Physiol A Neuroethol Sens Neural Behav Physiol 1998 182 737 746 10.1007/s003590050218 9631554

[B34] Colleye O Frederich B Vandewalle P Casadevall M Parmentier E Agonistic sounds in the skunk clownfish *Amphiprion akallopisos*: size-related variation in acoustic features J Fish Biol 2009 75 908 916 10.1111/j.1095-8649.2009.02316.x 20738587

[B35] Lobel PS Mann DA Spawning sounds of the damselfish, *Dascyllus albisella *(Pomacentridae), and relationship to male size Bioacoustics 1995 6 187 198

[B36] Connaughton MA Taylor MH Fine ML Effects of fish size and temperature on weakfish disturbance calls: implications for the mechanism of sound generation J Exp Biol 2000 203 1503 1512 10751166 10.1242/jeb.203.9.1503

[B37] Fine ML King CB Friel JP Loesser KE Newton S Sound production and locking of the pectoral spine of the channel catfish American Fisheries Society Symposium 1999 24 105 114

[B38] Ladich F Comparative analysis of swimbladder (drumming) and pectoral (stridulation) sounds in three families of catfishes Bioacoustics 1997 8 185 208

[B39] Schneider H Neuere Ergebnisse der Lautforschung bei Fischen Naturwissenschaften 1961 15 513 518 10.1007/BF00595330

[B40] Fine ML Friel JP McElroy D King CB Loesser KE Newton S Pectoral spine locking and sound production in the channel catfish *Ictalurus punctatus* Copeia 1997 1997 777 790 10.2307/1447295

[B41] Bosher BT Newton SH Fine ML The spine of the channel catfish, *Ictalurus punctatus*, as an anti-predator adaptation: an experimental study Ethology 2006 112 188 195 10.1111/j.1439-0310.2006.01146.x

[B42] Drescher O Meine Erlebnisse mit dem Marmorierten Fiederbartwels D Aqu u Terr Z (DATZ) 2007 60 16 20

[B43] Fuiman LA Poling KR Higgs DM Quantifying developmental progress for comparative studies of larval fishes Copeia 1998 1998 602 611 10.2307/1447790

[B44] Holcik J Banarescu P Evans D Holcik J General introduction to fishes The Freshwater Fishes of Europe 1989 1/II Wiesbaden: Aula Verlag 18 147

[B45] Wysocki LE Ladich F Effects of noise exposure on click detection and the temporal resolution ability of the goldfish auditory system Hear Res 2005 201 27 36 10.1016/j.heares.2004.08.015 15721558

[B46] Wysocki LE Ladich F Hearing in fishes under noise conditions JARO 2005 6 28 36 10.1007/s10162-004-4043-4 15735936 PMC2504637

[B47] Wysocki LE Ladich F Can fishes resolve temporal characteristics of sounds? New insights using auditory brainstem response Hear Res 2002 169 36 46 10.1016/S0378-5955(02)00336-2 12121738

[B48] Ladich F Wysocki LE Does speaker presentation affect auditory evoked potential thresholds in goldfish? Comp Biochem Physiol A Comp Physiol 2009 154 341 346 10.1016/j.cbpa.2009.07.004 19602445

[B49] Akamatsu T Okumura T Novarini N Yan HY Empirical refinements applicable to the recording of fish sounds in small tanks J Acoust Soc Am 2002 112 3073 3082 10.1121/1.1515799 12509030

[B50] Noll AM Cepstrum pitch detection J Acoust Soc Am 1967 41 293 309 10.1121/1.1910339 6040805

[B51] Amoser S Wysocki LE Ladich F Noise emission during the first powerboat race in an Alpine lake and potential impact on fish communities J Acoust Soc Am 2004 116 3789 3797 10.1121/1.1808219 15658729

